# Fc‐gamma receptor polymorphisms, cetuximab therapy, and overall survival in the CCTG CO.20 trial of metastatic colorectal cancer

**DOI:** 10.1002/cam4.1819

**Published:** 2018-10-14

**Authors:** Daniel Shepshelovich, Amanda R. Townsend, Osvaldo Espin‐Garcia, Lidija Latifovic, Chris J. O’Callaghan, Derek J. Jonker, Dongsheng Tu, Eric Chen, Eric Morgen, Timothy J. Price, Jeremy Shapiro, Lillian L. Siu, Michiaki Kubo, Alexander Dobrovic, Mark J. Ratain, Wei Xu, Taisei Mushiroda, Geoffrey Liu

**Affiliations:** ^1^ Division of Medical Oncology and Hematology, Princess Margaret Cancer Centre University of Toronto Toronto Ontario Canada; ^2^ Sackler School of Medicine Tel Aviv University Tel Aviv Israel; ^3^ Medical Oncology University of Adelaide Adelaide South Australia Australia; ^4^ Department of Biostatistics, Princess Margaret Cancer Centre, Dalla Lana School of Public Health University of Toronto Toronto Ontario Canada; ^5^ Epidemiology Division, Dalla Lana School of Public Health University of Toronto Toronto Ontario Canada; ^6^ Canadian Cancer Trials Group (CCTG) Queens University Kingston Ontario Canada; ^7^ Medical Oncology, The Ottawa Hospital University of Ottawa Ottawa Ontario Canada; ^8^ Department of Laboratory Medicine and Pathology, Mount Sinai Hospital University of Toronto Toronto Ontario Canada; ^9^ Department of Medical Oncology Cabrini Health Malvern Victoria Australia; ^10^ RIKEN Center for Integrative Medical Science Yokohama Japan; ^11^ Peter MacCallum Cancer Centre University of Melbourne Melbourne Victoria Australia; ^12^ Translational Genomics and Epigenomics Laboratory Olivia Newton‐John Cancer Research Institute Heidelberg Victoria Australia; ^13^ School of Cancer Medicine La Trobe University Bundoora Victoria Australia; ^14^ The University of Chicago Chicago Illinois; ^15^ Department of Medical Biophysics University of Toronto Toronto Ontario Canada

**Keywords:** cetuximab, FCGR2A, FCGR3A, polymorphism, survival

## Abstract

**Background:**

Two germ line Fc‐γ receptor (FCGR) polymorphisms, rs1801274 [*FCGR2A*; His(H)131Arg(R)] and rs396991 [*FCGR3A*; Phe(F)158Val(V)], produce altered proteins through amino acid substitutions. We previously reported that the *FCGR2A* H/H genotype was associated with longer overall survival (OS) in cetuximab‐treated chemotherapy‐refractory patients with metastatic colorectal cancer. Here, we aimed to replicate and extend this finding in the Canadian Clinical Trials Group CO.20 trial.

**Methods:**

After germ line DNA genotyping, polymorphic relationships with survival were assessed using log‐rank tests and hazard ratios (HR) from Cox proportional hazard models, adjusting for known prognostic factors. The dominant genetic inheritance model was used for the main analysis.

**Results:**

Of 592 wild‐type *KRAS* patients treated with cetuximab, those with the *FCGR2A* H/H genotype (n = 165, 28%) had improved OS (HR: 0.66, *P* < 0.001; median absolute benefit, 1.3 months) compared to those with R/‐ genotype (n = 427, 72%). Patients with H/R had intermediate results under a codominant genetic inheritance model (HR: 0.72, *P* = 0.003). No significant associations were found between *FCGR3A* genotype and OS. In an exploratory analysis, patients with the combination of *FCGR2A* H/H + *FCGR3A* F/F genotype had significantly better OS (HR: 0.33, *P* = 0.003; median absolute benefit, 12.5 months) than patients with the combination of double‐variant R/R + V/V genotype. Progression‐free survival results were similar to OS. Toxicity rates were not associated with either polymorphism.

**Conclusions:**

The *FCGR2A* genotype was associated with efficacy but not with toxicity in wild‐type *KRAS*, cetuximab‐treated colorectal cancer patients. *FCGR3A* genotype may modulate the relationship between *FCGR2A* polymorphism and outcome. *FCGR2A* is a promising biomarker for clinical management for these patients.

## 1 INTRODUCTION

Cetuximab is an IgG1 monoclonal antibody (mAb) that targets theepidermal growth factor receptor (EGFR) and has been shown to improve outcomes in patients with metastatic wild‐type *KRAS* colorectal carcinoma.^1^, ^2^ As many of these patients do not benefit from cetuximab treatment,^2^ there is an unmet need for additional predictive biomarkers, in addition to *RAS* and *BRAF* mutations.

One of cetuximab's mechanisms of action is antibody‐dependent cellular cytotoxicity (ADCC).^3^, ^4^ ADCC is initiated when the antigen‐binding fragment (Fab) binds to the tumor cell and the crystallizable fragment (Fc) binds to the crystallizable fragment gamma receptor (FCGR) on a natural killer cell, macrophage, or monocyte, creating a bridge from the tumor cell to the effector cell. Tumor cell recognition is then coupled with a lytic attack on the cancer cell mounted by effector cells.^5^, ^6^ Three classes of FCGR exist, encoded by related genes on the long arm of chromosome 1: *FCGR1*‐CD64; *FCGR2*‐CD32; and *FCGR3*‐CD16.^7^ Two polymorphisms located within coding regions of *FCGR2A* and *FCGR3A* were previously reported to be associated with the efficacy of cetuximab in colorectal cancer.^8^, ^9^ A nonsynonymous polymorphism in the extracellular domain of *FCGR2A* (rs1801274) changes the amino acid from histidine (H) to arginine (R), significantly reducing the receptor's affinity to Fc.^17^ The rs396991 polymorphism in *FCGR3A* is also found in the extracellular domain, leading either to a phenylalanine (F) or valine (V) substitution; this amino acid interacts with the lower hinge region of IgG1.^18^, ^19^ Previous studies of the association of these two polymorphisms with the efficacy of cetuximab reported mixed results.^8^, ^9^ Most of these studies had various limitations, including small sample size, non‐randomized patient selection, and suboptimal genotyping technique. A recent analysis of data from the Canadian Cancer Trials Group (CCTG) CO.17 randomized controlled trial found cetuximab treatment was associated with overall survival (OS) benefit in patients with metastatic wild‐type *KRAS* colorectal cancer who had the *FCGR2A* H/H genotype but not those with the R/‐ genotype. Patients with the H/R genotype had non‐statistically significant intermediate results.^20^ A *post hoc* analysis found cetuximab‐treated patients with the H/H genotype had longer OS than those with R/‐ genotype (univariate hazard ratio (HR) 0.63 (95% confidence interval (CI) 0.3‐0.9), adjusted HR: 0.57, 95% CI: 0.3‐0.8). This effect was not seen in the best supportive care arm. In contrast, no association was found between the *FCGR3A* polymorphism and any clinical outcome. The primary objective of this study was to replicate our previous finding of the association of *FCGR2A* polymorphism and OS in an independent, larger trial dataset after adjusting for other potential prognostic factors.

## 2 METHODS

### 2.1 Study design and population

This retrospective, secondary analysis of thegerm line polymorphisms *FCGR2A:H*→*R* (rs1801274, cytosine→thymine) and *FCGR3A:F*→*V* (rs396991, cytosine→adenine) in wild‐type *KRAS* patients used available DNA samples from the CCTG and the Australasian Gastro‐Intestinal Trials Group (AGITG) CO.20 trial.^21^ Briefly, this was a multicenter, open‐label, phase III randomized controlled trial; 750 chemotherapy‐refractory metastatic colorectal cancer patients were randomized (1:1) to cetuximab and placebo vs cetuximab and brivanib alaninate, a dual inhibitor of vascular endothelial growth factor receptor and fibroblast growth factor receptor tyrosine kinase.^22^ Three months after study initiation, the protocol was amended to enroll only patients with wild‐type *KRAS,* given new information regarding the lack of benefit of anti‐EGFR monoclonal antibodies in *KRAS* mutant colorectal cancer.^2^ Twenty‐one patients with mutated *KRAS* and four patients with indeterminable *KRAS* status were enrolled prior to the amendment. Our analysis was conducted on known wild‐type *KRAS* patients only. Patients in both arms received cetuximab intravenously at an initial loading dose of 400 mg/m^2^ over 120 minutes, followed by a weekly maintenance infusion of 250 mg/m^2^ over 60 minutes. Patients randomly assigned to the combination arm also received oral brivanib at 800 mg on a daily schedule. No significant difference in the primary outcome of OS was observed (8.8 months vs 8.1 months in the brivanib and the placebo groups, respectively, HR: 0.88, 95% CI: 0.74‐1.03; *P* = 0.12), despite a strong progression‐free survival (PFS) benefit favoring the experimental arm with both cetuximab and brivanib (HR: 0.72; 95% CI: 0.62‐0.84; *P* < 0.001).^21^

### 2.2 Outcomes

The primary objective of the current analysis was to evaluate the previously described association between *FCGR2A* polymorphism and OS in cetuximab‐treated patients. Exploratory objectives included the association of *FCGR2A* polymorphism and PFS and the associations of *FCGR3A* polymorphism and OS and PFS. OS was defined as the time from random assignment until death from any cause. PFS was defined as the time from random assignment until the first observation of disease progression or death from any cause. The CCTG trial database was used for all analyses. REMARK guidelines were followed.^23^ All outcomes were planned prior to analysis initiation.

### 2.3 DNA extraction and genotyping method

Whole blood samples from local sites were archived at the CCTG central tissue bank (Queen's University, Kingston, Canada). DNA was extracted using the Qiagen whole blood DNA Kit. DNA quantity (spectrophotometry) and quality (polymerase chain reactions) were checked.DNA was independently genotyped blindly in the laboratory of G. Liu using TaqMan assays ordered from Thermo Fisher scientific company (Waltham, MA, USA). Assay IDs were C_9077561_20 for rs1801274, and C_25815666_10 for rs396991, with a subset of 30 confirmed also by Sanger sequencing.^20^ Results were checked using Hardy‐Weinberg equilibrium testing.^24^ *KRAS* status was previously tested as a requirement for the CO.20 trial after the amendment.^21^

### 2.4 Statistical analysis

For the primary outcome analysis,*FCGR2A* polymorphism was compared with OS for all patients with genotyping results. Analyses were performed separately under the dominant, codominant, and additive genetic inheritance model assumptions; genotype was analyzed as a numerical variable for the additive model (number of copies of the minor allele), and as a categorical variable for the dominant and codominant models. The dominant genetic inheritance model was utilized for the primary analysis, consistent with our previous analysis.^20^ A formal power calculation for replication of our previous results under the dominant genetic inheritance model, assuming Hardy‐Weinberg equilibrium, found empirical power of 97.5% for the univariate HR and of 99.7% for the adjusted HR, at a significance level of 0.5%.^25^ For the exploratory analyses, *FCGR2A* polymorphism was compared with PFS, and *FCGR3A* polymorphism was compared with OS and PFS. Interaction between *FCGR2A* and *FCGR3A* polymorphisms was explored by comparing outcomes of patients with both H/H* FCGR2A and* F/F *FCGR3A* polymorphisms to those with the R/R and V/V polymorphisms and to the rest of the study cohort, as a recent meta‐analysis found the F/F *FCGR3A* genotype was associated with better OS.^12^ Additional exploratory analyses assessed potential relationships of *FCGR2A* and *FCGR3A* polymorphisms with toxicity. OS and PFS were assessed using Kaplan‐Meier curves, log‐rank tests (univariable analyses), and Cox proportional hazard models in multivariable analyses, adjusting for clinically relevant factors identified in the original trial analysis, including age, gender, side of primary tumor, stage, grade, number of metastatic sites, serum lactate dehydrogenase concentration, and treatment arm.^21^ Genomewide association studies (GWAS) generated a principal component analysis that was used to adjust for population stratification in the multivariable analyses. Tests for the assumption of proportional hazards were performed. R software version 3.3.1 (R Foundation for Statistical Computing, Vienna, Austria) was used for all analyses.

## 3 RESULTS

### 3.1 Patient and genotyping characteristics

**Figure 1:**
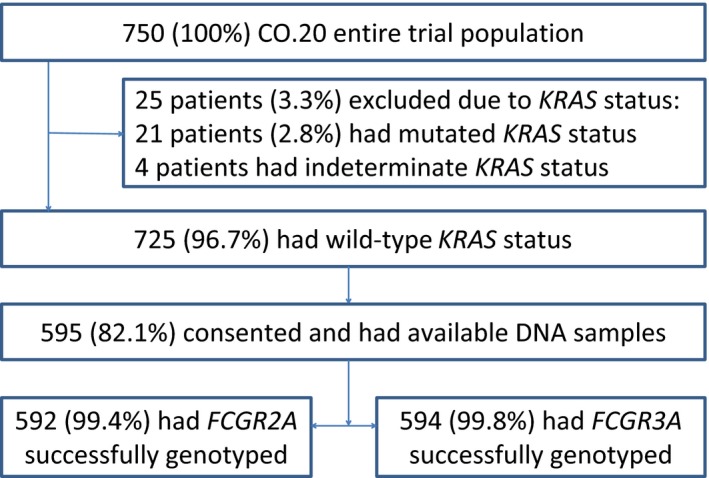
CONSORT diagram

The CO.20 trial included 725 patients who were known to be wild‐type *KRAS*, of whom 595 (82%) had provided consent for genotyping and had available DNA extracted from an EDTA‐treated whole blood sample. A total of 592 (99.4%) were successfully genotyped for *FCGR2A* and 594 (99.8%) for *FCGR3A*. The consort diagram of samples is shown in Figure 1. Demographic and disease variables are summarized in Table 1. A total of 165 patients (28%) had the H/H *FCGR2A* genotype, 299 (50%) had H/R, and 128 (22%) had R/R A total of 232 (39%) patients had the F/F *FCGR3A* genotype, 275 (46%) had F/V, and 87 (15%) had V/V. Both polymorphisms were in Hardy‐Weinberg equilibrium: *P* = 0.73 (*FCGR2A*) and *P* = 0.71 (*FCGR3A*). Linkage disequilibrium between *FCGR2A* and *FCGR3A* was low (*r* ^2 ^= 0.03, *D*′ = 0.25). Patient characteristics were not significantly associated with *FCGR2A* polymorphisms (Table 1) and were also comparable between the subsets of patients with or without genotyping data (Table S1). Distribution of *FCGR2A* and *FCGR3A* genotypes was comparable in the brivanib and control study arms (Table S2). There was 100% concordance between the TaqMan assays and the Sanger sequencing.

**Table 1:** Patient characteristics according to *FCGR2A* polymorphism

Characteristics	Genotyped patients (n = 592)	*FCGR2A* H/H (n = 165)	*FCGR2A* H/R (n = 299)	*FCGR2A* R/R (n = 128)	*P*‐value
Mean age, y (SD)	62.8 (10.7)	62.4 (11.1)	63 (10)	62.9 (12.1)	0.64
Male gender	392 (66%)	115 (70%)	190 (64%)	87 (68%)	0.37
Side of primary cancer					
Left	261 (44%)	74 (45%)	134 (45%)	53 (41%)	0.25
Right	125 (21%)	26 (16%)	66 (22%)	33 (26%)	
Rectal	206 (35%)	65 (39%)	99 (33%)	42 (33%)	
Tumor stage at initial diagnosis					
I/II	70 (12%)	28 (18%)	29 (10%)	13 (11%)	0.091
III	175 (31%)	52 (32%)	83 (29%)	40 (33%)	
IV	325 (57%)	80 (50%)	177 (61%)	68 (56%)	
Missing	22	5	10	7	
Tumor grade					
I	35 (6%)	9 (6%)	22 (8%)	4 (4%)	0.078
II	421 (78%)	128 (83%)	214 (77%)	79 (72%)	
III	86 (16%)	17 (11%)	42 (15%)	27 (25%)	
Missing	50	11	21	18	
Number of metastatic sites					
≤2	468 (79%)	135 (82%)	231 (77%)	102 (80%)	0.52
>2	124 (21%)	30 (18%)	68 (23%)	26 (20%)	
Number of previous lines of chemotherapy					
≤2	22 (4%)	6 (4%)	9 (3%)	7 (5%)	0.44
>2	570 (96%)	159 (96%)	290 (97%)	121 (95%)	

SD, standard deviation.

### 3.2 Association between FCGR2A polymorphisms and OS

**Figure 2:**
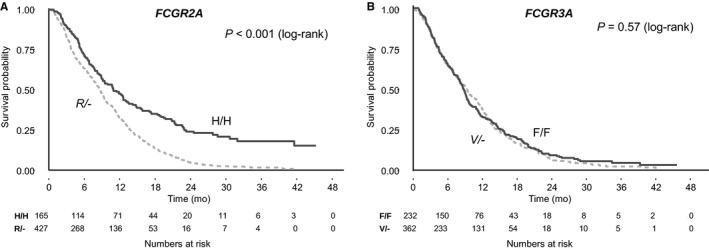
Kaplan‐Meier curves for overall survival by *FCGR2A* and *FCGR3A* polymorphisms under the dominant genetic model assumptions

**Figure 3:**
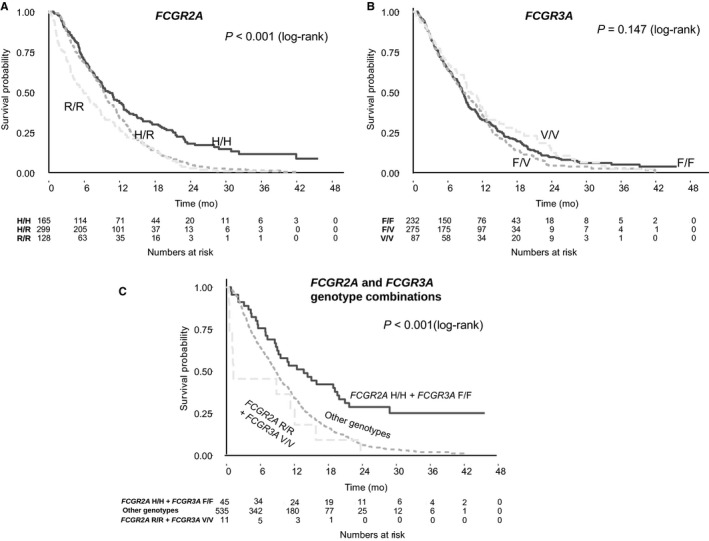
Kaplan‐Meier curves for overall survival by *FCGR2A* and *FCGR3A* polymorphisms under the codominant genetic model assumptions

All Cox models were consistent with the assumption of proportional hazards. There was a statistically significant association between *FCGR2A* genotype and OS in the study cohort (Table 2). In the primary univariable analysis under the dominant inheritance model, the H/H genotype was associated with better OS than the R/‐ genotype (HR: 0.61, 95% CI: 0.5‐0.74, *P* < 0.001, median absolute benefit 1.3 months; Figure 2). Similar results were obtained in the exploratory analyses under the additive model (OR: 0.71 per H allele, 95% CI: 0.63‐0.8, *P* < 0.001) and the codominant model, with the H/H genotype associated with better OS compared with the R/R genotype (HR: 0.51, 95% CI: 0.4‐0.65, *P* < 0.001, median absolute benefit 3.7 months), and intermediate outcomes for patients with the H/R genotype (HR: 0.65, 95% CI: 0.53‐0.8, *P* < 0.001; Figure 3). Results remained significant in multivariable analysis under all models (Table 2). There was no interaction between *FCGR2A* polymorphism and OS according to treatment arm (*P* = 0.37).

**Table 2:** Primary and exploratory multivariable analyses of the association between *FCGR2A* polymorphism and overall survival

Genetic inheritance model	Genotype	Median survival (mo)	One‐year survival (95% CI)	Two‐year survival (95% CI)	aHR (95% CI)	*P*‐value
Primary analysis						
Dominant	H/H	9.6	44% (37‐52)	18% (13‐26)	0.66 (0.54‐0.81)	<0.001
	R/‐	8.3	32% (27‐37)	4% (2‐8)	Reference	
Exploratory analyses						
Codominant	H/H	9.6	44% (37‐52)	18% (13‐26)	0.53 (0.41‐0.68)	<0.001
	H/R	9.3	34% (29‐40)	5% (3‐8)	0.72 (0.58‐0.89)	0.003
	R/R	5.9	27% (21‐36)	3% (1‐8)	Reference	
Additive	H/H	9.6	44% (37‐52)	18% (13‐26)	Per increase in 1 wild‐type	<0.001
	H/R	9.3	34% (29‐40)	5% (3‐8)	H allele: 0.72 (0.64‐0.83)	
	R/R	5.9	27% (21‐36)	3% (1‐8)		

Analyses are adjusted for clinically relevant factors identified in the original trial analysis.^21^ Genomewide association studies (GWAS), used for population stratification, were available for 566 of the 592 (95.6%) patients with genotyping results, and subset analyses of these 566 patients that included adjustment by principal components found virtually identical results.

aHR, adjusted hazard ratio from Cox proportional hazards models; CI, confidence interval.

### 3.3 Exploratory associations between FCGR polymorphisms and clinical outcomes

There was a statistically significant association between *FCGR2A* genotype and PFS in the study cohort. In univariable analysis under the dominant inheritance model, the H/H genotype was associated with better PFS compared with the R/‐ genotype (HR: 0.80, 95% CI: 0.67‐0.95, *P* = 0.01, median absolute benefit 1.6 months). Similar results were obtained under the additive inheritance model (OR: 0.80 per H allele, 95% CI: 0.71‐0.9, *P* < 0.001) and under the codominant model, with the H/H genotype associated with better PFS than patients with the R/R genotype (HR: 0.63, 95% CI: 0.5‐0.8, *P* < 0.001, median absolute benefit 3.3 months) and similar PFS compared with the H/R genotype (HR: 0.87, 95% CI: 0.71‐1.05, *P* = 0.14; Figure S1). Results remained significant in multivariable analysis under all models (Table 3). There was no interaction between *FCGR2A* polymorphism and PFS according to treatment arm (*P* = 0.65).

**Table 3:** Exploratory multivariable analysis of the associations between the *FCGR2A* and *FCGR3A* polymorphisms and clinical outcomes

Polymorphism	Genotype	Median survival (mo)	Codominant model: aHR (95% CI), *P*‐value	Additive model: aHR (95% CI), *P*‐value	Dominant model: aHR (95% CI), *P*‐value
Overall survival					
*FCGR3A*	F/F	8.6	1.03 (0.78‐1.35), 0.84	Per increase in 1 wild‐type F allele: 0.99 (0.87‐1.12), 0.80	F/F vs V/‐: 0.93 (0.78‐1.11), *P* = 0.44
	F/V	8.8	0.91 (0.75‐1.09), 0.30		
	V/V	9.5	reference		
*FCGR2A* and *FCGR3A* genotype combinations	H/H + F/F[Link]	13.7	0.33 (0.16‐0.68), 0.003	Not applicable	Not applicable
	Others	8.7	0.52 (0.36‐0.75), <0.001		
	R/R + V/V[Link]	1.2	Reference		
Progression‐free survival					
*FCGR2A*	H/H	5.2	0.65 (0.51‐0.83), <0.001	Per increase in 1 wild‐type H allele: 0.81 (0.72‐0.92), <0.001	H/H vs R/‐: 0.80 (0.66‐0.97), *P* = 0.02
	H/R	3.7	0.87 (0.71‐1.05), 0.15		
	R/R	1.9	Reference		
*FCGR3A*	F/F	3.7	0.99 (0.76‐1.28), 0.92	Per increase in 1 wild‐type F allele: 0.97 (0.86‐1.1), 0.64	F/F vs V/‐: 0.93 (0.78‐1.1), *P* = 0.39
	F/V	3.6	0.91 (0.76‐1.09), 0.31		
	V/V	5.1	Reference		
*FCGR2A* and *FCGR3A* genotype combinations	H/H + F/F[Link]	5.5	0.45 (0.22‐0.92), 0.03	Not applicable	Not applicable
	Others	3.6	0.79 (0.58‐1.09), 0.15		
	R/R + V/V[Link]	1.0	Reference		

Analyses are adjusted for clinically relevant factors identified in the original trial analysis.^21^ Genomewide association studies (GWAS), used for population stratification, were available for 566 of the 592 (95.6%) patients with genotyping results, and subset analyses of these 566 patients that included adjustment by principal components found virtually identical results.

aHR, adjusted hazard ratio from Cox proportional hazards models; CI, confidence interval.

Double wild‐type genotype (wild type for both *FCGR2A* and *FCGR3A*).

Double‐homozygous variant genotype (homozygous variant for both *FCGR2A* and *FCGR3A*).

No association was observed between *FCGR3A* polymorphism and OS (Table 3, Figures 2 and 3) or PFS (Table 3, Figure S1). However, when combinations of genotypes of *FCGR2A* and *FCGR3A* polymorphisms were compared with OS and PFS both in univariable analysis and adjusted for key known prognostic factors, patients with both the H/H *FCGR2A* and the F/F* FCGR3A* double wild‐type genotypes had significantly better OS compared to those with the R/R and V/V polymorphisms (HR: 0.33, 95% CI: 0.16‐0.68, *P* = 0.003; median absolute benefit, 12.5 months) and those with any other genotype (Table 3, Figure 3).

### 3.4 Exploratory association between FCGR polymorphisms and toxicity

There was no association between *FCGR2A* (*P* = 0.13) or *FCGR3A* (*P* = 0.64) and any grade 3 or greater treatment toxicity in multivariable analysis. A pre‐specified analysis of the interaction between *FCGR2A* and *FCGR3A* polymorphisms and skin rash also found no statistically significant interaction, *P* = 0.20 and *P* = 0.71, respectively. Exploratory analyses further found virtually identical relationships in subset analyses by trial arm.

## 4 DISCUSSION

We have successfully replicated our previous results demonstrating an association between *FCGR2A* genotype and survival in wild‐type *KRAS* colorectal cancer patients treated with cetuximab. H/H genotype was associated with significantly longer OS than the R/‐ genotype under the dominant genetic model assumptions. Additional exploratory analyses under the additive and codominant genetic inheritance models found intermediate results for patients with the heterozygous genotype; this finding expands on the prior study which had too small a sample size to adequately assess these models. The similar and consistently significant results under the additive and codominant genetic inheritance models imply that the clinical benefit for colorectal cancer patients treated with cetuximab could be additive in nature, with an improvement of approximately ~30% in survival benefit for every H allele of *FCGR2A*.

The large sample size was also used to explore the joint effect of both *FCGR2A* and *FCGR3A* polymorphisms together; we found that the best outcomes were in patients with the double wild‐type genotypes and the worst were found in the 2% of patients with the double‐homozygous variant genotypes, with a threefold difference in OS (absolute median difference 12.5 months) after adjustment for other prognostic factors, although confidence intervals were wide. As the presence of both homozygous variants is a rare occurrence, the additional clinical utility of this finding is limited. However, it provides further insight into the mechanistic role of *FCGR* polymorphisms in clinical outcomes of cetuximab‐treated, metastatic colorectal cancer, namely, the role of *FCGR3A* polymorphism in modifying the primary relationship between *FCGR2A* and clinical outcomes.

Regardless, the primary clinical implication of this study relates to the common variant allele frequency of the *FCGR2A* polymorphism^26^: OS was twice longer for patients with the *FCGR2A* H/H polymorphism than those with the R/R genotype, corresponding to median absolute difference of 3.7 months in the patient population now known to benefit from cetuximab, namely *KRAS* wild‐type patients. Of additional importance is that genotypes associated with better OS were not associated with higher rates of grade 3 or greater toxicity, nor were they related to skin toxicity. This is likely due to the different mechanisms involved: Toxicity is thought to be mediated through competitive inhibition of the EGFR receptor,^27^, ^28^ a different mechanism of action from Fc‐γ receptor‐regulated ADCC.^3^, ^4^ No interaction was found between treatment arm and the analyzed polymorphisms, which was to be expected as both treatment arms included the same cetuximab regimen, and brivanib efficacy was not expected to be affected by FCGR polymorphisms.

Although our findings replicate the results of several prior publications^8^, ^14^, ^15^ and of our previous analysis,^20^ others have reported different results. Notably, a recent consortium analysis of 660 cetuximab‐treated patients with wild‐type *KRAS* metastatic colorectal carcinoma did not find any association between *FCGR2A* polymorphisms and clinical outcome.^16^ However, the study population was heterogeneous in regard to the studied intervention, with a minority of the patients treated with single‐agent cetuximab, and most patients treated with irinotecan‐based regimens that included cetuximab.^16^ Also, only approximately a third of the patients had available germ line DNA from blood samples, with the rest of the patients having putatively normal DNA extracted from formalin‐fixed paraffin‐embedded (FFPE) slides,^16^ an approach that has been demonstrated to be unreliable for at least some genes.^30^ Both of our studies were conducted in relatively homogenous and prospective patient populations with germ line DNA genotyped from blood samples, and the results replicated.^23^

While the *FCGR2A* wild‐type genotype was associated with better OS and PFS, this study's results were of greater magnitude for OS, consistent with our previous findings in CO.17,^20^ and with several large studies of anti‐EGFR mAbs for wild‐type *KRAS* colorectal cancer, such as the FIRE‐3 and the PEAK trials.^31^, ^32^ Although OS can be confounded by post‐trial treatments, it is less likely to be affected by pre‐trial confounders, given the randomized nature of these trials. An alternative explanation might be a joint effect between *FCGR* polymorphisms and anti‐EGFR mAbs, untested in the aforementioned trials and identified in this trial, causing failure of surrogacy of PFS for OS.

Similar interactions between *FCGR2A* polymorphism and clinical outcomes have been reported for other mAbs and malignancies. Patients with germ line H/H polymorphism treated with the anti‐human epidermal growth factor receptor 2 (HER2) mAb trastuzumab for early HER2‐positive breast cancer were more likely to achieve pathological response while those with metastatic disease had better objective response rate and PFS.^33^, ^34^ The H/H polymorphism of *FCGR2A* was also associated with better response to anti‐TNF mAbs in patients with rheumatoid arthritis.^36^, ^37^ This evidence further supports a genetic role in determining cetuximab treatment outcomes.

Limitations of this study include an inability to genotype the entire sample as the blood sample component of the CO.20 trial was voluntary. However, the genotyped subgroup represented 79% of the entire trial population, with similar clinical and demographic characteristics (Table S1). Wild‐type *KRAS* status was defined based on the assessment of lack of mutations seen in exon 2; the effect of other, significantly rarer *RAS* and *BRAF* mutations on this polymorphism‐outcome association, was not assessed in this analysis.^40^ EGFR polymorphisms previously reported to be associated with cetuximab efficacy and toxicity were also not assessed.^41^, ^42^ Finally, although we could perform joint analyses of combinations of *FCGR2A* and *FCGR3A* genotypes, we still had an insufficient sample size to perform a formal interaction analysis.

In conclusion, this analysis confirms that *FCGR2A* H/H polymorphism in patients with wild‐type *KRAS* metastatic colorectal cancer treated with cetuximab is associated with significantly longer OS without affecting toxicity profiles. This association may be modified by the *FCGR3A* polymorphism*,* as patients with the double wild‐type genotypes of H/H and F/F genotypes had the best clinical outcomes. This replication in a large, separate dataset provides evidence to evaluate prospectively the utility of *FCGR* polymorphisms as biomarkers for clinical management in this patient population.

## CONFLICT OF INTEREST

The authors declare no potential conflict of interests.

## References

[1] Jonker DJ , O'Callaghan CJ , Karapetis CS , et al. Cetuximab for the treatment of colorectal cancer. N Engl J Med. 2007;357:2040‐2048. 18003960 10.1056/NEJMoa071834

[2] Karapetis CS , Khambata‐Ford S , Jonker DJ , et al. K‐RAS mutations and benefit from cetuximab in advanced colorectal cancer. N Engl J Med. 2008;359(17):1757‐1765. 18946061 10.1056/NEJMoa0804385

[3] Mellor JD , Brown MP , Irving HR , Zalcberg JR , Dobrovic A . A critical review of the role of Fc gamma receptor polymorphisms in the response to monoclonal antibodies in cancer. J Hematol Oncol. 2013;6:1. 23286345 10.1186/1756-8722-6-1 PMC3549734

[4] Weiner LM , Surana R , Wang S . Monoclonal antibodies: versatile platforms for cancer immunotherapy. Nat Rev Immunol. 2010;10:317‐327. 20414205 10.1038/nri2744 PMC3508064

[5] Alderson KL , Sondel PM . Clinical cancer therapy by NK cells via antibody dependent cell‐mediated cytotoxicity. J Biomed Biotechnol. 2011;2011:379123. 21660134 10.1155/2011/379123 PMC3110303

[6] Ravetch JV , Bolland S . IgG Fc receptors. Annu Rev Immunol. 2001;19:275‐290. 11244038 10.1146/annurev.immunol.19.1.275

[7] Gessner JE , Heiken H , Tamm A , Schmidt RE . The IgG Fc receptor family. Ann Hematol. 1998;76:231‐248. 9692811 10.1007/s002770050396

[8] Bibeau F , Lopez‐Crapez E , Di Fiore F , et al. Impact of Fc‐gamma‐RIIa‐Fc‐gamma‐RIIIa polymorphisms and KRAS mutations on the clinical outcome of patients with metastatic colorectal cancer treated with cetuximab plus irinotecan. J Clin Oncol. 2009;27:1122‐1129. 19164213 10.1200/JCO.2008.18.0463

[9] Dahan L , Norguet E , Etienne‐Grimaldi MC , et al. Pharmacogenetic profiling and cetuximab outcome in patients with advanced colorectal cancer. BMC Cancer. 2011;11:496. 22117530 10.1186/1471-2407-11-496 PMC3235081

[10] Etienne‐Grimaldi MC , Bennouna J , Formento JL , et al. Multifactorial pharmacogenetic analysis in colorectal cancer patients receiving 5‐fluorouracil‐based therapy together with cetuximab‐irinotecan. Br J Clin Pharmacol. 2012;73:776‐785. 22486600 10.1111/j.1365-2125.2011.04141.x PMC3403205

[11] Zhang W , Gordon M , Schultheis AM , et al. FCGR2A and FCGR3A polymorphisms associated with clinical outcome of epidermal growth factor receptor expressing metastatic colorectal cancer patients treated with single‐agent cetuximab. J Clin Oncol. 2007;25:3712‐3718. 17704420 10.1200/JCO.2006.08.8021

[12] Ying HQ , Wang F , Chen XL , et al. FCGR2A, FCGR3A polymorphisms and therapeutic efficacy of anti‐EGFR monoclonal antibody in metastatic colorectal cancer. Oncotarget. 2015;6(29):28071‐28083. 26363448 10.18632/oncotarget.4872 PMC4695045

[13] Paez D , Pare L , Espinosa I , et al. Immunoglobulin G fragment C receptor polymorphisms and KRAS mutations: are they useful biomarkers of clinical outcome in advanced colorectal cancer treated with anti‐EGFR‐based therapy? Cancer Sci. 2010;101:2048‐2053. 20550522 10.1111/j.1349-7006.2010.01621.x PMC11158139

[14] Rodriguez J , Zarate R , Bandres E , et al. Fc gamma receptor polymorphisms as predictive markers of Cetuximab efficacy in epidermal growth factor receptor downstream‐mutated metastatic colorectal cancer. Eur J Cancer. 2012;48:1774‐1780. 22305465 10.1016/j.ejca.2012.01.007

[15] Kjersem JB , Skovlund E , Ikdahl T , et al. FCGR2A and FCGR3A polymorphisms and clinical outcome in metastatic colorectal cancer patients treated with first‐line 5‐fluorouracil/folinic acid and oxaliplatin+/‐ cetuximab. BMC Cancer. 2014;14:340. 24884501 10.1186/1471-2407-14-340 PMC4045863

[16] Geva R , Vecchione L , Kalogeras KT , et al. FCGR polymorphisms and cetuximab efficacy in chemorefractory metastatic colorectal cancer: an international consortium study. Gut. 2015;64:921‐928. 25011934 10.1136/gutjnl-2014-307234

[17] Warmerdam PA , van de Winkel JG , Vlug A , Westerdaal NA , Capel PJ . A single amino acid in the second Ig‐like domain of the human Fc gamma receptor II is critical for human IgG2 binding. J Immunol. 1991;147:1338‐1343. 1831223

[18] Radaev S , Motyka S , Sautes‐Fridman C , Fridman WH , Sun PD . The structure of a human type III Fcgamma receptor in complex with Fc. J Biol Chem. 2001;276:16469‐16477. 11297532 10.1074/jbc.M100350200

[19] Sondermann P , Huber R , Oosthuizen V , Jacob U . The 3.2‐A crystal structure of the human IgG1 Fc fragment‐Fc gammaRIII complex. Nature. 2000;406:267‐273. 10917521 10.1038/35018508

[20] Liu G , Tu D , Lewis M , et al. Fc‐γ receptor polymorphisms, cetuximab therapy, and survival in the NCIC CTG CO.17 trial of colorectal cancer. Clin Cancer Res. 2016;22(10):2435‐2444. 27179112 10.1158/1078-0432.CCR-15-0414

[21] Siu LL , Shapiro JD , Jonker DJ , et al. Phase III randomized, placebo‐controlled study of cetuximab plus brivanib alaninate versus cetuximab plus placebo in patients with metastatic, chemotherapy‐refractory, wild‐type K‐RAS colorectal carcinoma: the NCIC Clinical Trials Group and AGITG CO.20 Trial. J Clin Oncol. 2013;31(19):2477‐2484. 23690424 10.1200/JCO.2012.46.0543

[22] Huynh H , Ngo VC , Fargnoli J , et al. Brivanib alaninate, a dual inhibitor of vascular endothelial growth factor receptor and fibroblast growth factor receptor tyrosine kinases, induces growth inhibition in mouse models of human hepatocellular carcinoma. Clin Cancer Res. 2008;14(19):6146‐6153. 18829493 10.1158/1078-0432.CCR-08-0509

[23] McShane LM , Altman DG , Sauerbrei W , et al. Reporting recommendations for tumor marker prognostic studies. J Clin Oncol. 2005;23:9067‐9072. 16172462 10.1200/JCO.2004.01.0454

[24] Rodriguez S , Gaunt TR , Day IN . Hardy‐Weinberg equilibrium testing of biological ascertainment for Mendelian randomization studies. Am J Epidemiol. 2009;169(4):505‐514. 19126586 10.1093/aje/kwn359 PMC2640163

[25] Owzar K , Li Z , Cox N , Jung SH . Power and sample size calculations for SNP association studies with censored time‐to‐event outcomes. Genet Epidemiol. 2012;36(6):538‐548. 22685040 10.1002/gepi.21645 PMC3592339

[26] NCBI SNP database. https://www.ncbi.nlm.nih.gov/snp/?term=rs1801274. Accessed August 24, 2017.

[27] Lacouture ME . Mechanisms of cutaneous toxicities to EGFR inhibitors. Nat Rev Cancer. 2006;6(10):803‐812. 16990857 10.1038/nrc1970

[28] Belum VR , Fontanilla Patel H , Lacouture ME , Rodeck U . Skin toxicity of targeted cancer agents: mechanisms and intervention. Future Oncol. 2013;9(8):1161‐1170. 23902247 10.2217/fon.13.62

[29] Holcmann M , Sibilia M . Mechanisms underlying skin disorders induced by EGFR inhibitors. Mol Cell Oncol. 2015;2(4):e1004969. 27308503 10.1080/23723556.2015.1004969 PMC4905346

[30] Goetz MP , Sun JX , Suman VJ , et al. Loss of heterozygosity at the CYP2D6 locus in breast cancer: implications for germline pharmacogenetic studies. J Natl Cancer Inst. 2014;107(2):pii: dju401. 10.1093/jnci/dju401 PMC4565524 25490892

[31] Heinemann V , von Weikersthal LF , Decker T , et al. FOLFIRI plus cetuximab versus FOLFIRI plus bevacizumab as first‐line treatment for patients with metastatic colorectal cancer (FIRE‐3): a randomised, open‐label, phase 3 trial. Lancet Oncol. 2014;15(10):1065‐1075. 25088940 10.1016/S1470-2045(14)70330-4

[32] Schwartzberg LS , Rivera F , Karthaus M , et al. PEAK: a randomized, multicenter phase II study of panitumumab plus modified fluorouracil, leucovorin, and oxaliplatin (mFOLFOX6) or bevacizumab plus mFOLFOX6 in patients with previously untreated, unresectable, wild‐type KRAS exon 2 metastatic colorectal cancer. J Clin Oncol. 2014;32(21):2240‐2247. 24687833 10.1200/JCO.2013.53.2473

[33] Musolino A , Naldi N , Bortesi B , et al. Immunoglobulin G Fragment C receptor polymorphisms and clinical efficacy of trastuzumab‐based therapy in patients with HER‐2/neu‐positive metastatic breast cancer. J Clin Oncol. 2008;26:1789‐1796. 18347005 10.1200/JCO.2007.14.8957

[34] Tamura K , Shimizu C , Hojo T , et al. FcgammaR2A and 3A polymorphisms predict clinical outcome of trastuzumab in both neoadjuvant and metastatic settings in patients with HER2‐positive breast cancer. Ann Oncol. 2011;22:1302‐1307. 21109570 10.1093/annonc/mdq585

[35] Roca L , Diéras V , Roché H , et al. Correlation of HER2, FCGR2A, and FCGR3A gene polymorphisms with trastuzumab related cardiac toxicity and efficacy in a subgroup of patients from UNICANCER‐PACS 04 trial. Breast Cancer Res Treat. 2013;139(3):789‐800. 23780683 10.1007/s10549-013-2587-x

[36] Cañete JD , Suárez B , Hernández MV , et al. Influence of variants of Fc gamma receptors IIA and IIIA on the American College of Rheumatology and European League Against Rheumatism responses to anti‐tumour necrosis factor alpha therapy in rheumatoid arthritis. Ann Rheum Dis. 2009;68(10):1547‐1552. 18930989 10.1136/ard.2008.096982

[37] Avila‐Pedretti G , Tornero J , Fernández‐Nebro A , et al. Variation at FCGR2A and functionally related genes is associated with the response to anti‐TNF therapy in rheumatoid arthritis. PLoS One. 2015;10(4):e0122088. 25848939 10.1371/journal.pone.0122088 PMC4388501

[38] Dávila‐Fajardo CL , van der Straaten T , Baak‐Pablo R , et al. FcGR genetic polymorphisms and the response to adalimumab in patients with rheumatoid arthritis. Pharmacogenomics. 2015;16(4):373‐381. 25823785 10.2217/pgs.14.178

[39] Montes A , Perez‐Pampin E , Narváez J , et al. Association of FCGR2A with the response to infliximab treatment of patients with rheumatoid arthritis. Pharmacogenet Genomics. 2014;24(5):238‐245. 24667440 10.1097/FPC.0000000000000042

[40] Douillard JY , Oliner KS , Siena S , et al. Panitumumab‐FOLFOX4 treatment and RAS mutations in colorectal cancer. N Engl J Med. 2013;369(11):1023‐1034. 24024839 10.1056/NEJMoa1305275

[41] Gonçalves A , Esteyries S , Taylor‐Smedra B . A polymorphism of EGFR extracellular domain is associated with progression free‐survival in metastatic colorectal cancer patients receiving cetuximab‐based treatment. BMC Cancer. 2008;8:169. 18544172 10.1186/1471-2407-8-169 PMC2432064

[42] Graziano F , Ruzzo A , Loupakis F , et al. Pharmacogenetic profiling for cetuximab plus irinotecan therapy in patients with refractory advanced colorectal cancer. J Clin Oncol. 2008;26(9):1427‐1434. 18349392 10.1200/JCO.2007.12.4602

[43] Inoue Y , Hazama S , Iwamoto S , et al. FcγR and EGFR polymorphisms as predictive markers of cetuximab efficacy in metastatic colorectal cancer. Mol Diagn Ther. 2014;18(5):541‐548. 24828248 10.1007/s40291-014-0103-6

