# Supplementary figures and images for: Fc‐gamma receptor polymorphisms, cetuximab therapy, and overall survival in the CCTG CO.20 trial of metastatic colorectal cancer
Source: Cancer Med. 2018 Oct 14;7(11):5478–87. doi: 10.1002/cam4.1819 (PMC6246957; doi:10.1002/cam4.1819)

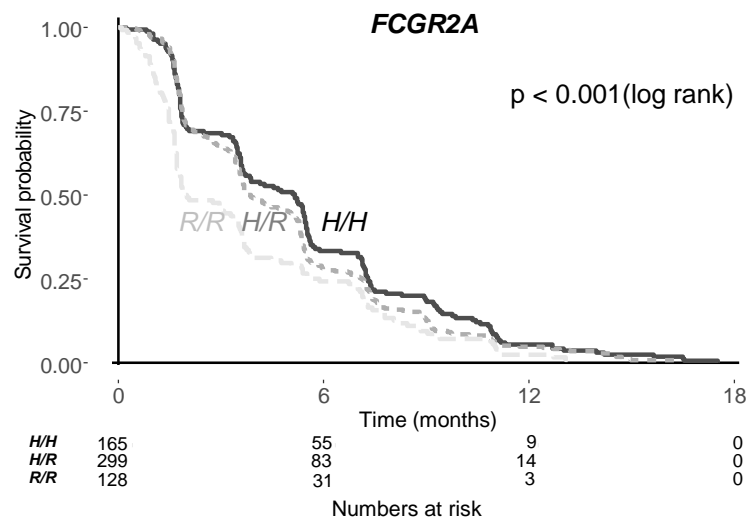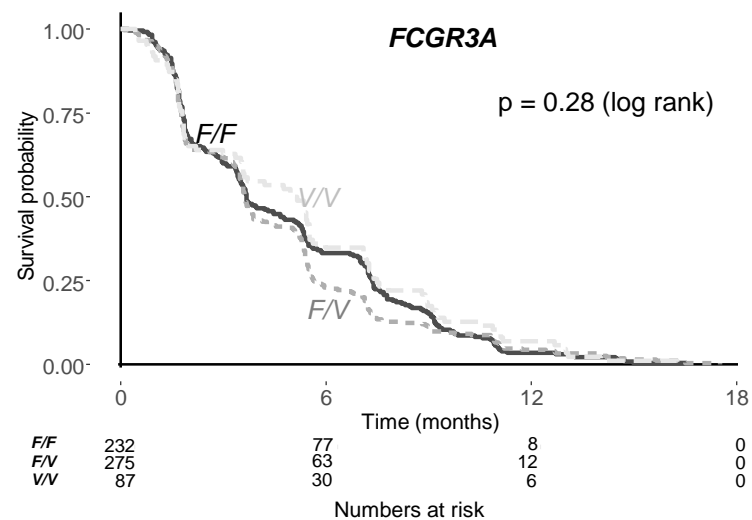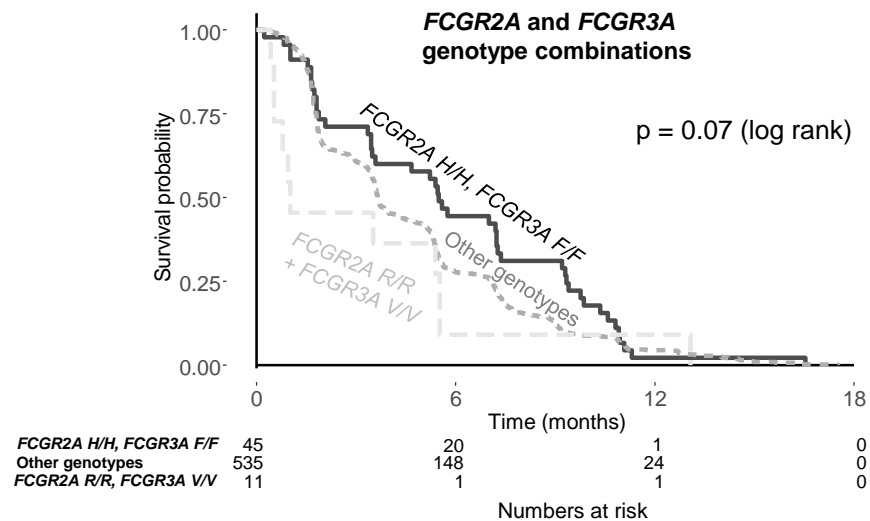

Supplement: Supplementary file 1 [file CAM4-7-5478-s001.pdf]
