# Supplementary material for: Fc‐gamma receptor polymorphisms, cetuximab therapy, and overall survival in the CCTG CO.20 trial of metastatic colorectal cancer
Source: Cancer Med. 2018 Oct 14;7(11):5478–87. doi: 10.1002/cam4.1819 (PMC6246957; doi:10.1002/cam4.1819)
Supplement: Supplementary file 2 [file CAM4-7-5478-s002.docx]

**Table 1S: Characteristics of all *K-RAS* wild type patients versus patients with or without genotyping results**

| **Characteristic** | **All *K-RAS* wild-type patients (n=725)** | **Genotyped patients (n=592)** | **Non-genotyped patients (n=133)** | **p-value** |
| --- | --- | --- | --- | --- |
| Mean age, years (SD) | 62.6 (10.7) | 62.8 (10.7) | 61.7 (10.7) | 0.29 |
| Male gender | 468 (65%) | 392 (66%) | 76 (57%) | 0.06 |
| Side of primary cancer  Left  Right  Rectal | 318 (44%)  159 (22%)  248 (34%) | 261 (44%)  125 (21%)  206 (35%) | 57 (43%)  34 (25%)  42 (32%) | 0.50 |
| Tumor stage at initial diagnosis  I/II  III  IV  Missing | 94 (14%)  217 (31%)  386 (55%)  28 | 70 (12%)  175 (31%)  325 (57%)  22 | 24 (19%)  42 (33%)  61 (48%)  6 | 0.08 |
| Tumor grade  I  II  III  Missing | 43 (6%)  515 (78%)  103 (16%)  64 | 35 (6%)  421 (78%)  86 (16%)  50 | 8 (7%)  94 (79%)  17 (14%)  14 | 0.93 |
| Number of metastatic sites  ≤2  >2 | 568 (78%)  157 (22%) | 468 (79%)  124 (21%) | 100 (75%)  33 (25%) | 0.35 |
| Number of previous lines of chemotherapy  ≤2  >2 | 28 (4%)  697 (96%) | 22 (4%)  570 (96%) | 6 (5%)  127 (95%) | 0.62 |
| Best clinical response:  Progressive disease  Partial response  Stable disease  Indeterminable | 220 (30%)  80 (11%)  358 (50%)  67 (9%) | 183 (31%)  69 (12%)  291 (49%)  49 (8%) | 37 (28%)  11 (8%)  67 (50%)  18 (14%) | 0.56 |
| Mean overall survival, months (SD) | 10.5 (8.1) | 10.6 (8.1) | 9.9 (7.9) | 0.37 |
| Mean progression free survival, months (SD) | 4.9 (3.8) | 4.8 (3.5) | 5.4 (5.0) | 0.07 |
| Any grade III/IV toxicity | 526 (73%) | 425 (72%) | 101 (76%) | 0.39 |

SD: standard deviation
