# Supplementary material for: Fc‐gamma receptor polymorphisms, cetuximab therapy, and overall survival in the CCTG CO.20 trial of metastatic colorectal cancer
Source: Cancer Med. 2018 Oct 14;7(11):5478–87. doi: 10.1002/cam4.1819 (PMC6246957; doi:10.1002/cam4.1819)
Supplement: Supplementary file 3 [file CAM4-7-5478-s003.docx]

**Table 2S: Distribution of FCGR genotypes in control and study arms**

| **Genotype** | **Study cohort** | **Brivanib arm (n=365)** | **Control arm (n=360)** | **p** |
| --- | --- | --- | --- | --- |
| **FCGR2A** | | | | |
| H/H | 165 (28%) | 81 (27%) | 84 (28%) | 0.78 |
| H/R | 299 (50%) | 153 (52%) | 146 (49%) |  |
| R/R | 128 (22%) | 61 (21%) | 67 (23%) |  |
| Missing^1^ | 133 | 70 | 63 |  |
| **FCGR3A** | | | | |
| F/F | 232 (39%) | 119 (40%) | 113 (38%) | 0.48 |
| F/V | 275 (46%) | 130 (44%) | 145 (49%) |  |
| V/V | 87 (15%) | 47 (16%) | 40 (13%) |  |
| Missing^1^ | 131 | 69 | 62 |  |

^1^Patients with no available DNA or no consent to genotyping.
